# Strain Field Mapping of Dislocations in a Ge/Si Heterostructure

**DOI:** 10.1371/journal.pone.0062672

**Published:** 2013-04-23

**Authors:** Quanlong Liu, Chunwang Zhao, Shaojian Su, Jijun Li, Yongming Xing, Buwen Cheng

**Affiliations:** 1 College of Science, Inner Mongolia University of Technology, Hohhot, China; 2 College of Information Science and Engineering, Huaqiao University, Xiamen, China; 3 State Key Laboratory on Integrated Optoelectronics, Institute of Semiconductors, Chinese Academy of Sciences, Beijing, China; Boston College, United States of America

## Abstract

Ge/Si heterostructure with fully strain-relaxed Ge film was grown on a Si (001) substrate by using a two-step process by ultra-high vacuum chemical vapor deposition. The dislocations in the Ge/Si heterostructure were experimentally investigated by high-resolution transmission electron microscopy (HRTEM). The dislocations at the Ge/Si interface were identified to be 90° full-edge dislocations, which are the most efficient way for obtaining a fully relaxed Ge film. The only defect found in the Ge epitaxial film was a 60° dislocation. The nanoscale strain field of the dislocations was mapped by geometric phase analysis technique from the HRTEM image. The strain field around the edge component of the 60° dislocation core was compared with those of the Peierls–Nabarro and Foreman dislocation models. Comparison results show that the Foreman model with *a* = 1.5 can describe appropriately the strain field around the edge component of a 60° dislocation core in a relaxed Ge film on a Si substrate.

## Introduction

Dislocation, which is a classic topic in material science because of its significance in the mechanical property of materials, has received considerable attention from researchers [1]–[3]. Aside from mechanical properties, the electrical properties of materials, including their electrical conductivity and electron mobility, are affected by the deformation fields of dislocations in semiconductors such as Ge [4]. Thus, Ge dislocations should undergo quantitative deformation analysis to control the electrical properties of Ge when used in an integrated circuit or in heterostructure devices [5]. However, the deformation field around a dislocation core is at the nanoscale. Performing a nanoscale strain analysis on dislocations was a very difficult task due to the lack of experimental techniques with a sufficient accuracy and spatial resolution before. Recently, a method combining high-resolution transmission electron microscopy (HRTEM) and geometric phase analysis (GPA) has shown to be an effective method for mapping strain fields at the nanoscale. GPA [6] is an image processing technique that has been used in a wide variety of systems, including Si heterostructures [7], nanoparticles [8], crack tips [9], and so on. Numerical Moiré can be calculated from the geometric phase of the GPA. The numerical Moiré pattern acts as a lens that magnifies not only the lattice spacing but also the deformation. By selecting a geometric phase image, a desired numerical Moiré image that corresponds to a group of special crystal planes can be obtained, thus allowing a detailed analysis of this group of crystal planes [10].

Dislocation models have had no powerful experimental proof because of lack of experimental deformation field measurements. Nevertheless, many theoretical predictions and dislocation models are available. The Peierls–Nabarro (P–N) model [11]–[12], which has been discussed by many researchers [13]–[14], is one of the most important dislocation models available. However, the width of a dislocation calculated by the P–N model is about 1.5 atomic spacings if the Poisson’s ratio *v* ∼ 0.3, which is too small for actual crystals. Foreman proposed an improved model based on the P–N model [15]. In the Foreman model, the P–N model is extended to a dislocation family of changeable widths by introducing factor *a*. In this model, *a* = 4 was fitted for bubble raft experiment and was considered equivalent to the P–N model when *a = *1. The P–N model has recently been reported as the most appropriate theoretical model for describing the strain fields around a pure-edge dislocation core in gold [16]. The Foreman model with *a* = 3 describes the strain field of threading dislocations in CeO_2_ thin films better [17]. Most recently, the Foreman model with *a* = 1.5 describes the strain fields around an edge dislocation core in graphene better [18]. However, the most appropriate dislocation model for Ge remains unknown. Therefore, this study presents a nanoscale strain mapping for Ge/Si heterostructures. Particularly, the measured strain field around the edge component of a 60° dislocation core in Ge film was compared with that of the P–N and Foreman models. This paper aims to identify the type of dislocations in Ge/Si heterostructures and to verify experimentally the currently available dislocation models.

### Strain of Edge Dislocation Described by the P–N Model

According to the P–N model, the strain of a dislocation along the *x* direction is written as
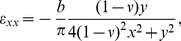
(1)where *x* and *y* are the right-angle coordinates centered on the dislocation core position, *b* is the magnitude of the Burgers vector of a dislocation, and *v* is the Poisson’s ratio. The width of a dislocation in the P–N model is 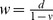, where *d* is the lattice constant [11], [12].

### Strain of Edge Dislocation Described by the Foreman Model

According to the Foreman model, the strain of a dislocation along the *x* direction can be written as
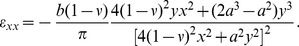
(2)


Here, the P–N model is extended to a family of dislocations with greater widths by introducing the factor *a*. Equation 2 will be reduced to Equation 1 when *a* = 1. The width of a dislocation in the Foreman model is 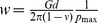, where *G* is the shear modulus, and *p*_max_ is the maximum shear stress that decreases as *a* (i.e., dislocation width) increases.

### Geometric Phase Analysis

An HRTEM image formed at the crystal zone axis is considered a set of interference fringes corresponding to the atomic planes of a specimen. These interference fringes were analyzed individually by GPA to extract the deformation information. Inverse Fourier transform was conducted on the masked original HRTEM image. The data type of the inverse Fourier transform image is complex. Thus, the local geometric phase was obtained by calculating the arctangent of the imaginary component divided by the real component. The geometric phase *P* ***_g_***(***r***) of these local Fourier components is directly related to the displacement field component ***u***(***r***) in the direction of the reciprocal lattice vector ***g***. *P* ***_g_***(***r***) is calculated through the following equation:
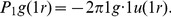
(3)


The 2D displacement fields are determined by measuring the two-phase images, namely, *P_g_*_1_(***r***) and *P_g_*_2_(***r***), as follows:
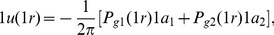
(4)where ***a***_1_ and ***a***_2_ are the basis vectors of the lattices in real space that correspond to the reciprocal lattices defined by ***g***_1_ and ***g***_2_, respectively [6]. Equation (4) is presented in matrix form as follows:



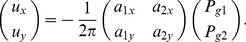
(5)Thus, the plane strain is written as
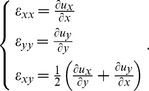
(6)


## Materials and Methods

### Ge Epitaxial Film Preparation

A custom-designed ultra-high vacuum chemical vapor deposition system equipped with pyrolytic BN effusion cells was used to grow Ge on a Si(001) substrate. The system used had a base pressure of 3×10^−8^ Pa. In situ reflection high-energy electron diffraction (RHEED) was used to monitor wafer growth. Before growing the Ge film, the wafers were cleaned by using the Radio Corporation of America (RCA) method. The wafers were then degassed in a pre-treatment chamber at 400°C and then baked in a growth chamber at 930°C for deoxidation. The Ge film was grown by using a two-step process. In the first step, the substrate was kept at 200°C to grow a 40 nm, flat, relaxed epitaxial Ge film. At such low temperature, 3D Ge islands nucleated and the strain was released via formation of misfit dislocations. In the second step, the substrate temperature was increased to 500°C to lower the dislocation density, thus obtaining good crystalline quality [19].

### Transmission Electron Microscopy

The TEM sample was prepared for cross-sectional imaging along the [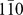] direction by using a standard technique that involves mechanical grinding followed by ion milling. The HRTEM experiment was performed by using a JEM-2010 TEM at 200 kV. The images were then recorded by using a Gatan 1k×1k slow-scan charge-coupled device and then processed by using GPA Phase, a software program developed in the Gatan DigitalMicrograph environment.

## Results and Discussion

**Figure 1:**
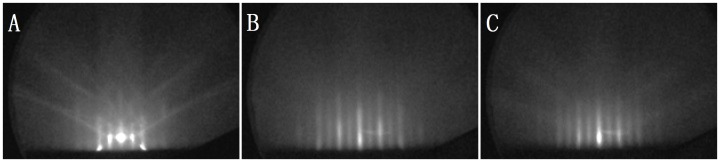
In situ RHEED patterns during the growth process of a Ge/Si heterostructure. (A) RHEED pattern of deoxidized Si substrate before growth, (B) RHEED pattern of the Ge epitaxial film grown at 200°C, (C) RHEED pattern of the Ge epitaxial film grown at 500°C.

Figure 1 shows the in situ RHEED patterns during the growth process, and Figure 1A shows the RHEED pattern of the deoxidized Si substrate before growth. A well-developed 2×1 reconstruction and Kikuchi lines can be observed. After the 40 nm Ge buffer layer was grown at 200°C, the RHEED pattern (Figure 1B) presented a 2×1 reconstruction with no Kikuchi lines. This result indicates that the surface was flat and that the Stranski–Krastanov-related 2D to 3D transitions was successfully suppressed. After the temperature was increased to 500°C (Figure 1C), the wafer growth maintained a 2D (layer-by-layer) mode. In addition, small spotty patterns and Kikuchi lines progressively appeared, thus indicating high crystallinity [20].

**Figure 2:**
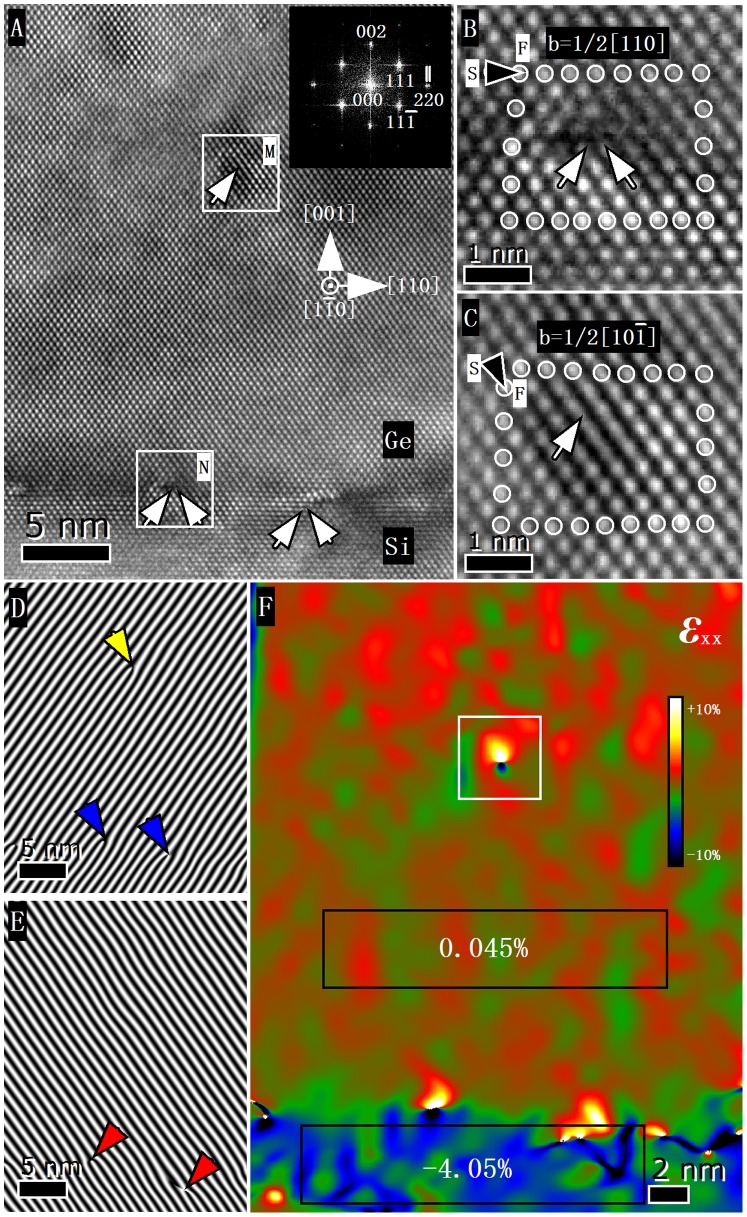
HRTEM image and strain field of the Ge/Si heterostructure. (A) HRTEM image and FFT pattern (inset) of a Ge/Si heterostructure, (B) Burgers circuit around the 90° full-edge dislocation, (C) Burgers circuit around the 60° dislocation, (D) 3× numerical Moiré pattern of a (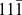) plane, (E) 3× numerical Moiré image of (111) planes, (F) the strain field of Figure 2(A) calculated by using GPA.

Figure 2A depicts a cross-sectional HRTEM image of the Ge/Si heterostructure. The inset in Figure 2A shows the fast Fourier transform (FFT) pattern of the HRTEM image. The diffraction spots in the FFT image were divided into paired separate spots (indicated by two white lines for the paired spots of 220), which correspond to those of the Si and Ge crystals. The distances from the 000 spot to the paired separate spots of 220 in the FFT pattern were calculated. Calculation results show that the differences in the lattice constant between Si and Ge are about 4%. This distance is close to the Ge–Si lattice mismatch, thus indicating that the lattice mismatch strain of the Ge films is almost relaxed [20]. No stacking faults and V-shaped defects were found in the Ge epitaxial film, which shows that the Ge epitaxial film has high quality. The main defect observed in this experiment is that the misfit dislocations were mostly confined in the Ge/Si interface. The characters of these misfit dislocations can be directly determined from the HRTEM images by drawing a Burgers circuit around the dislocations. An example of the misfit dislocation (boxed area N in Figure 2A) is enlarged and shown in Figure 2B, where the sense vector points into the page, and the direction of the Burgers circuit is clockwise according to the right–hand/finish–start convention [21]. The Burgers vector of the misfit dislocation is identified as 1/2 [110] (Figure 2B), which is a 90° full-edge dislocation [22] that is formed by the reaction of two 60° dislocations (marked by white arrows) according to the following dislocation reaction: 1/2[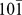](111)+1/2[011](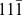) = 1/2[110](001) [23]. These 90° full-edge dislocations are sessile dislocations, and the Burgers vectors are parallel to the Ge/Si interface (001). The primary slip plane of Ge and Si is {111}. The Burgers vectors of these dislocations do not lie in the primary slip plane, so they are immobile. These 90° full-edge dislocations represent the missing half-planes in Si, relax the Ge film to achieve lattice matching.

A single dislocation (boxed area M in Figure 2A), whose core is located at a distance of 19 nm from the Ge/Si interface, is the only defect that can be found in the Ge film. The Ge film deposited at 200°C has thickness of 40 nm. Therefore, the dislocation is observed in the Ge film deposited at 200°C. It is well known that for diamond cubic lattice systems, such as Ge and Si, the Burgers vector of initial dislocations are 1/2<101>, and the dislocations lie in the {111} glide planes of Ge or Si. These dislocations are called 60° dislocations because the Burgers vector is at a 60° angle with the sense vector [23]. Thus, the Burgers vectors of this dislocation can be identified to be 1/2[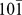] (Figure 2C). Given that the atomic arrangements of the 60° dislocation were projected onto the HRTEM image (Figure 2C), the Burgers vector that is decided by the current Burgers circuit in Figure 2C is the edge component that corresponds to the Burgers vector 1/2[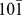] in the projection plane [24]. The extra half-plane is located at the (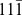) plane, and the Burgers vector lies in the (111) plane. The edge component (*b_e_*) can be decided to be 1/4[].

To visualize the atomic arrangement of these dislocations, the numerical Moiré images for the two {111} planes have been calculated by using a magnification factor of three, as shown in Figures 2D and 2E. The edge component *b_e_* of the 60° dislocation in the Ge film is inclined at 54.44° to the interface, and only one extra half-plane exists (marked by a yellow arrow in Figure 2D). Two extra half-planes exist (marked by blue arrows in Figure 2D and red arrows in Figure 2E) in the projection plane of the atomic arrangement of the 90° dislocation. The two extra half-planes of the atomic arrangement of the 90° dislocations are (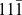) and (111), which have a symmetric arrangement around the core in a 90° dislocation.

Taking the *x*-axis parallel to [110], and the *y*-axis parallel to [001], then the strain field can be calculated from the HRTEM image (Figure 2A), as shown in Figure 2F. The color scale indicates strain changes of –10% to +10%. The mean of strain is 0.045% in the black line boxed area in the Ge film and the mean of strain is –4.05% in the boxed area in the Si substrate. Thus, the strain is near zero in the Ge film, except in the convergence regions around the 60° dislocation. The lattice constant of Si, *d*_Si_ = 0.5431 nm, is approximately 4% smaller than that of Ge, *d*_Ge_ = 0.5657 nm. The measured misfit strain is in good agreement with the lattice mismatch of Ge and Si, indicating that the Ge film deposited at 200°C is fully relaxed. Some convergence regions of strain at the Ge/Si interface are present, in which these regions correspond to the misfit dislocations.

**Figure 3:**
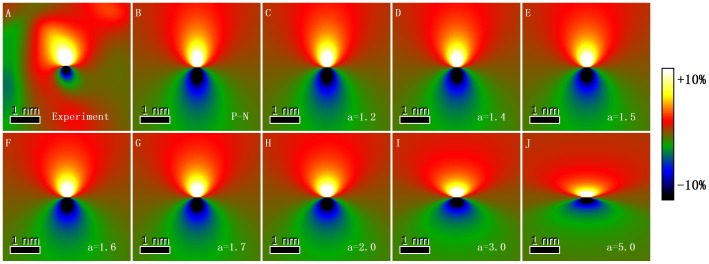
Experimental and theoretical strain fields around the edge component of a 60° dislocation core. (A) experimental strain field, (B) strain field by P–N model, (C)–(J) strain fields by Foreman model with *a* = 1.2, 1.4, 1.5, 1.6, 1.7, 2.0, 3.0, and 5.0, respectively.

To verify whether the strain field of the edge component of a 60° dislocation can be described by a dislocation model, the main strain field around the edge component of the 60° dislocation core (white line boxed area in Figure 2F) was enlarged, as shown in Figure 3A. The size of the selected area is 4.45 nm × 4.45 nm. Figures 3B to 3J show the theoretical strain fields calculated using the Poisson’s ratio (*ν*_Ge_ = 0.25) [25], the projected Burgers vector (*b_e_* = 0.3464 nm), and the origin situated at the dislocation core by the P–N and Foreman models with different values of *a*. A convergence region of strain exists around the dislocation core. The strains are positive and tensile in the upper half region, whereas the lattice is compressive on the lower half because of the extra half-plane. The largest strain values occur in the immediate core region, and the strain far from the dislocation core is small. Qualitatively, the experimental strain field and the theoretical strain fields do not exhibit much apparent difference around the dislocation core. The strain maps are all eight-shaped. However, the eight-shaped strain maps become wider and shorter and the dislocation width increases when the factor *a* of the Foreman model increases.

The F-test has been employed to determine the optimum dislocation model that can describe the experimental strain field [26]. In the F-test, two images are analyzed whether they have equal variances and whether the experimental and theoretical models agree. We have tested the null hypothesis that the population variances for the experiment and the theoretical model are equal. Null hypothesis is defined as 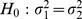 and the alternative hypothesis is defined as 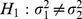, where 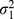 and 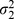 are respectively the population variances for the experiment and the theoretical model. Test statistic *F* is defined as 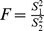, where 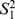 is the sample variance in the experiment and 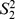 is the sample variance in the theoretical model. Table 1 shows the calculated *F* for the experiment and for the Foreman model with different values of *a*. *F_a_*_ = 1.0_ corresponds to the P–N model. The value of *F* is shown to increase gradually with increasing *a*. The size of these strain maps is *N* = 128 × 128 = 16384, as shown in Figure 3. Thus, the critical values of the *F* distribution for a two-tailed test are 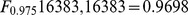 and 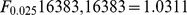 at the 0.05 significance level. The value of *F* calculated from the Foreman model with *a* = 1.5 and *a* = 1.6 can satisfy the equation 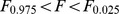. However, *F_a_*_ = 1.5_ is closer to the ideal value of 1.0000 than *F_a_*_ = 1.6_ is. The F-test indicates that not enough evidence is available to reject the null hypothesis *H*_0_ and that the variances in the experimental and theoretical results by the Foreman model with *a* = 1.5 are equal at the 0.05 significance level. Therefore, according to the F-test analysis results, the Foreman model with *a* = 1.5 is the most appropriate dislocation model for describing the strain field around the edge component of a 60° dislocation core in a relaxed Ge film on a Si substrate.

**Table 1:** Calculated *F* for the experiment and Foreman model with different values of *a*.

*a*	1.0	1.2	1.4	1.5	1.6	1.7	2.0	3.0	5.0
*F*	0.8913	0.9186	0.9620	0.9883	1.0167	1.0465	1.1628	1.5277	2.3184

This table shows the statistical results from the F-test.

### Conclusions

A high-quality Ge epitaxial film was successfully grown on a Si (001) substrate by using a two-step process by ultra-high vacuum chemical vapor deposition. The Ge epitaxial film was fully relaxed by the 90° full-edge dislocations located at the Ge/Si interface. The nanoscale strain field of the Ge/Si heterostructure was mapped using geometric phase analysis technique from the HRTEM image. The strain field around the edge component of a 60° dislocation core, which is only defect in the Ge epitaxial film, was compared with those of the P–N and Foreman dislocation models with different alterable factor *a*. On the basis of the F-test analysis, the Foreman model with *a* = 1.5 was identified to be the most appropriate dislocation model for describing the strain field around the edge component of a 60° dislocation core in a relaxed Ge film on a Si substrate.
